# Effect of etchant containing an Urushiol monomer from lacquer sap on dentin biostability and bonding performance

**DOI:** 10.3389/fbioe.2023.1251655

**Published:** 2023-10-13

**Authors:** Ying Zhao, Xuanwen Xu, Lu Li, Kai Zheng, Xiaoqian Wang, Ming Zhang, Yan Xu

**Affiliations:** ^1^ Jiangsu Province Key Laboratory of Oral Diseases, Nanjing Medical University, Nanjing, China; ^2^ Department of Prosthodontics, The Affiliated Stomatological Hospital of Nanjing Medical University, Nanjing, China; ^3^ Jiangsu Province Engineering Research Center of Stomatological Translational Medicine, Nanjing, China; ^4^ Department of Periodontics, The Affiliated Stomatological Hospital of Nanjing Medical University, Nanjing, China

**Keywords:** dentin biostability, oral microecology, universal adhesive, MMPs inhibition, urushiol

## Abstract

**Objectives:** This study aimed to evaluate the effectiveness of urushiol as an additive to surface acid etchant on dentin structure, by assessing the biostability of dentin, and determine the bonding strengths of dentin and enamel to the composite in the complicated oral microecology.

**Methods:** Etchants with different concentrations of urushiol (0.5, 1, or 3 wt%) were formulated and tested for their bonding performance. Demineralized dentin beams that were etched with experimental etchants were incubated in simulated body fluid solutions by evaluating the weight decrement after 1 month. The effects of urushiol on dentin and matrix metalloproteinases were confirmed by scanning electron microscopy (SEM). Moreover, the antibiotic actions of urushiol on the common cariogenic bacteria *Streptococcus mutans*, *Streptococcus sanguinis*, and *Streptococcus gordonii* as well as the biofilm were evaluated, and its effect on bacterial morphology was observed by scanning electron microscopy. Finally, enamel and dentin specimens were prepared from human molars to determine the depth of demineralization by the etchants and the relationship with the resin bond strengths to enamel and dentin (μTBS) and the morphology of the bonding interface.

**Results:** Urushiol could interact with dentine and inhibit collagenase activity, resulting in biostable dentine. The application of the etchants containing 0.5, 1, or 3 wt% urushiol significantly improved the durability of the dentin bonding interface with its instinctive antibacterial property (*p* < 0.05).

**Conclusion:** Urushiol not only improves dentin stability by interacting with collagen and inactivating MMP activity but also plays a role in the antibacterial effects in the complicated oral microecology. The effectiveness of urushiol etchant prolongs the longevity of bonded dental restorations without compromising clinical operation time.

## 1 Introduction

Dentin bonding has been a challenge ever since it became a clinical procedure due to the complex structure of the dentin matrix, such as the internal wettability of dentin (Van Meerbeek et al., 2011; Demarco et al., 2017) and collagen fibers that are easily degraded by endogenous enzymes (Hass et al., 2016). Regarding the self-etch or etch-and-rinse modes, universal adhesive is an alternative that increases choice (Van Meerbeek et al., 2020). There is increasing evidence that the achievement of universal adhesives varies on the basis of application mode (Hong et al., 2021). Their underperformance in self-etch mode not only *in vitro* but *in vivo* studies has shown reductions in durability, poor marginal fits, and greater staining as dentin is not etched (Matos et al., 2020; Stape et al., 2021). However, the discrepancy between the depths of dentin after etching and adhesive penetration leads to the exposure of collagen fibers in the hybrid layer. The biostability of dentin decreases, and these unwrapped collagen fibers are prone to hydrolysis and enzymatic degradation (Nakabayashi et al., 1982; Perdigao et al., 2013). Moreover, in the complicated oral microecology, secondary caries in the interface between the tooth and restoration is one of the main causes of repair failure (Opdam et al., 2007).

As the concept of dental caries etiology has been continuously studied, the homeostasis between commensal and pathogenic bacteria has drawn growing attention. Currently, dental caries is caused by an imbalanced microecology and multispecies biofilms formed by pathogenic bacteria rather than simplified species (Takahashi and Nyvad, 2011). Therefore, studying the characteristics of multispecies biofilms is of great significance. Notwithstanding, many previous studies only evaluated the effects of antibacterial materials on *Streptococcus mutans*, with only a few studies taking multispecies biofilms into account (Song et al., 2020; Zhang et al., 2020). *Streptococcus* *sanguinis* is a pioneer colonist of oral bacteria, usually fixing onto them (Zhao et al., 2019), and is considered an oral commensal species (Kreth et al., 2005). Therefore, this study investigated the multispecies biofilms formed by *S. mutans*, *S. sanguinis*, and *Streptococcus gordonii* to simulate the microecology of oral biofilms *in vivo*.

Over the years, adhesive systems have undergone substantial improvements, including their chemical properties, interactions with dentin matrices, and clinical protocols (Saikaew et al., 2016). The latest progress in dentin bonding has attempted to reduce clinical procedures and technology sensitivity, likewise developing strategies to extend the lifespan of bonding interfaces (Hass et al., 2021). Counteractive measures consist of improving the resistance of resin ingredients to hydrolysis, inhibiting the biodegradation of collagen fiber, and adding antibacterial components to prevent the harm caused by bacteria (Breschi et al., 2010).

Colleagues first referred the action of dentin matrix metalloproteinases (MMPs) on a series of endogenous peptidases causing decomposition of dentin substrate in carious lesions (Hannas et al., 2007). At present, the use of collagen crosslinking agents and MMP inhibitors for biological modification of dentin seems to be a promising strategy that can prolong the resistance of the bonding interface (Yi et al., 2019). However, their application for pretreatment significantly increases chair-side time. To avoid adding more steps during the bonding process, attempts have been made to add the bioactive ingredients described above to the adhesive or etchant. It is crucial to achieve sufficient hybridization and durability of the adhesion interface within collagen fibers (Matuda et al., 2016).

The strong adhesion ability demonstrated by ancient Buddhist statues in humid environments over thousands of years has provided an impetus for exploring novel materials to improve the stability of dentin bonding (Wu et al., 2018). The natural renewable product inferred to above is lacquer sap, which mostly consists of urushiol (Lu et al., 2006). The current application of urushiol in the dentistry mainly focuses on its antibacterial properties (Cha and Shin, 2016). Our previous studies have confirmed that it not only effectively promotes collagen crosslinking as a primer but also improves the hydrophobicity of the adhesive system (Zhao et al., 2021; Zhao et al., 2022). In this context, the purpose of this study was to evaluate urushiol’s effects on dentin biostability and cariogenic multispecies biofilms formed by *S.mutans*, *S.sanguinis*, and *S.gordonii* for its application as a novel etchant. Moreover, to investigate the effect of the simplified universal adhesive combined with urushiol etchant application, its physicochemical properties, bioactive functions, and bonding performances were tested.

## 2 Materials and methods

### 2.1 Materials

The extracted urushiol was provided by Wuhan National Lacquer Co., Ltd. (Wuhan, China). Phosphoric acid and dimethyl sulfoxide (DMSO) were purchased from Solabio Co. Ltd. (Shanghai, China). *S. mutans* ACTT 25175 (Maryland, United States), *S. sanguinis* ATCC 10556 and *S. gordonii* ATCC 10558 were cultured for 48 h in brain-heart infusion (BHI) broth from QingDaoHopebio-Technology Co., Ltd. (Qingdao, China) supplemented with 5% sucrose at 37°C with 5% CO_2_. Resin composite Filtex Z350XT and Adper universal Adhesive were obtained from 3 M (St. Paul, United States). The EnzChek™ Gelatinase/Collagenase Assay Kit was purchased from Invitrogen (Carlsbad, United States).

### 2.2 Preparation of the etchant

**FIGURE 1:**
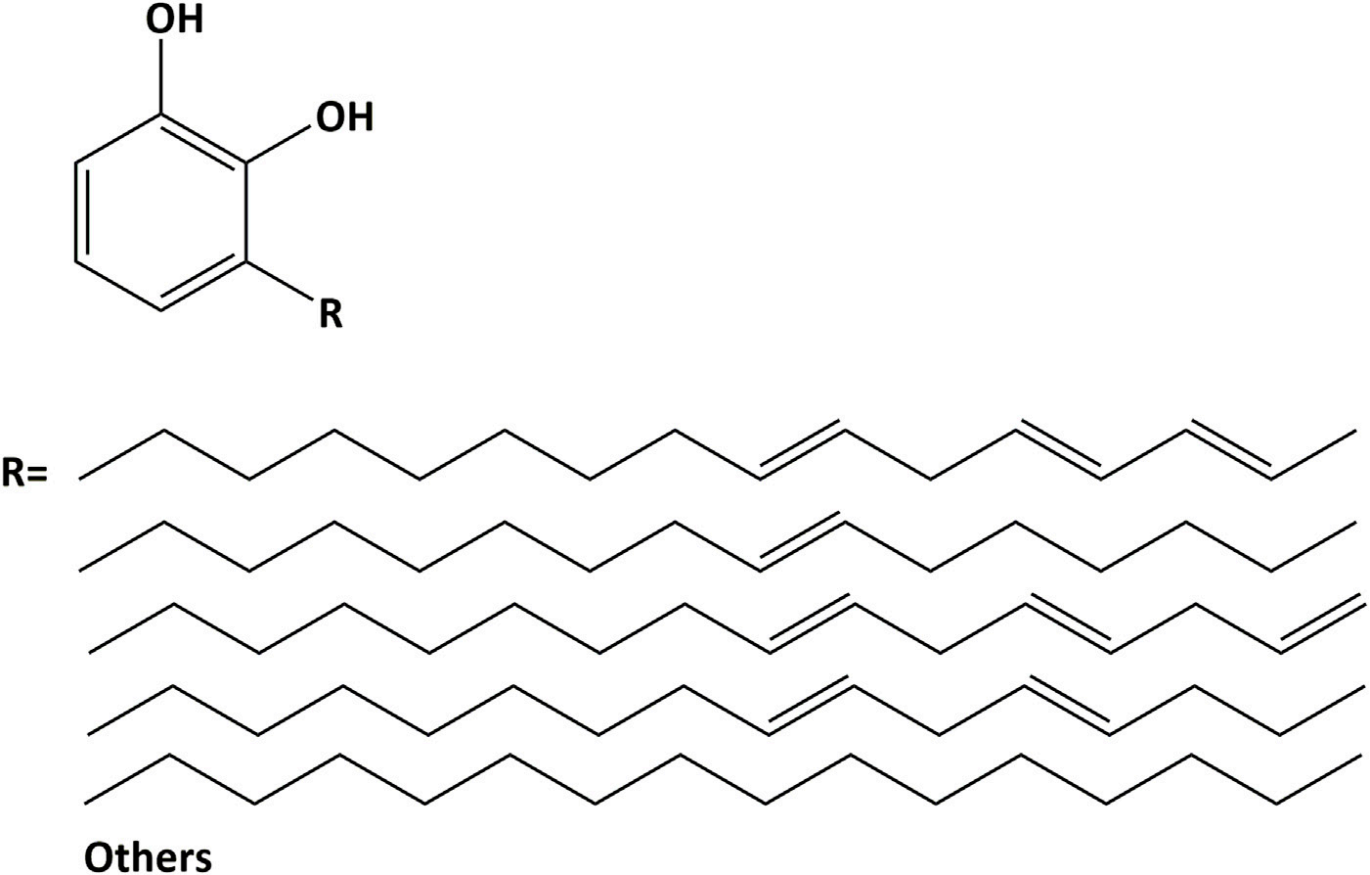
The most abundant component of lacquer sap. The chemical structure of urushiol.

Urushiol (Figure 1) was dissolved in DMSO solutions. After complete dissolution, 85% phosphoric acid and deionized water were added, generating biomodified etchants containing 10 wt% phosphoric acid, 10 wt% DMSO, and urushiol at concentrations of 0.5, 1, or 3 wt%. Commercial phosphoric acid gel was used as a control. The pH values of the etchants were measured using a pH meter.

### 2.3 Preparation of teeth

Fifty caries-free human third molars were obtained from patients who provided informed consent under a protocol approved by the Affiliated Stomatological Hospital of Nanjing Medical University. Extracted teeth were stored in 0.96% (w/v) phosphate buffered saline containing 0.002% sodium azide at 4°C and used for experiments within 1 month. Dentin beams with dimensions as slabs and beams of 1 × 2 × 6 mm^3^ (for dentin experiments) or 1 × 1 × 6 mm^3^ (for the other tests) were cut from the mid-coronal dentin by a low-speed diamond saw under cool water.

To determine that there was no residual enamel, the dentin surface was observed using a stereoscopical microscope. A standardized smear layer was achieved by placing 600-grit wet SiC paper under flowing water.

### 2.4 Dentine biostability

#### 2.4.1 Molecular docking test

Molecular docking was used to forecast the binding mode of small molecules and proteins, and docking was accomplished using AutoDock Vina 1.1.21 software. Before docking, the crystal structure of the target protein was obtained from the PDB database (PDB codes were 4oy5, 1cgd, and 1qsu). The three-dimensional framework of the target small molecule was constructed by ChemDraw and converted to a three-dimensional structure by Chem3D, and the energy of the small molecule was minimized by using the mmff94 force field.

Before formal docking, PyMOL 2.5 software 1 protein was used for preparation, including dehydrogenation and the removal of water molecules and non-ligand small molecules. The docking box was then defined to locate the docking position of the protein. Then, small molecules and receptor proteins in PDB format were converted to pdbqt format using adfrsuite 1.02. Finally, docking was carried out. During docking, the conformation search detail was set to 32, and the other parameters remained consistent with the default settings. The conformation with the highest affinity score was output as the correct conformation, which was visually analyzed using PyMOL 2.5.

#### 2.4.2 Collagenase biodegradation resistance

To determine the effect of urushiol on dentin biostability over time, dentin beams were etched by different groups as above for 48 h (n = 10 beams/group). After treatment, dentin beams were rinsed in deionized water for 24 h at 4°C and dried at room temperature in a desiccator for 24 h. The dry weight of the beams (W_1_) was tested by a digital balance, and the beams were rehydrated in deionized water followed by immersion in 1 ml of simulated body fluid (SBF) at 37°C for 30 days with the effects of endogenous proteases. Subsequently, dentin beams were dried in a desiccator again, and dry weight (W_2_) was tested as described above. The degradation of dentin substrate was confirmed by the percentage of change in dry weight before and after the 30-day incubation period.

### 2.5 Antibacterial activity of Urushiol

#### 2.5.1 Cultivation of bacteria

The antimicrobial efficacy of urushiol was evaluated against *S. mutans, S. sanguinis*, and *S. gordonii*, which are common caries-related bacteria. Each kind of bacteria was cultivated in a constant temperature shaker at 37°C ± 1°C and 130 r/min for 24 h. The concentration of bacteria was obtained by recording the OD value at 600 nm. The initial concentration of the three bacteria was 1 × 10^9^ CFU/ml-5×10^9^ CFU/ml.

#### 2.5.2 Growth curve test

To investigate the performance of urushiol at different concentrations on bacteria, a growth curve was formed. A bacterial suspension with BHI was used as a control; urushiol was prepared with final concentrations of 0.5, 0.25, 0.125, 0.0625, 0.03125, 0.015625, and 0.0078125 mg/ml, and the concentration of the bacterial solution was 10^6^ CFU/ml. An aliquot was transferred every 4 h from each culture starting from 0 h until 48 h.

#### 2.5.3 Minimum inhibitory concentration (MIC) test and minimum bactericidal concentration (MBC)

The minimum inhibitory concentration (MIC) was founded on the maximum extract concentration with the least number of colonies. The minimum extract concentration with sterile colony growth was the minimum bactericidal concentration (MBC). The MIC was confirmed in light of the instructions of a standard susceptibility broth dilution technique. Solutions of *S. mutans, S. sanguinis*, and *S. gordonii* were diluted in a 96-well plate with fresh BHI at 10^5^ CFU/ml. The suspension of bacteria with BHI served as the control. After cultivation in a constant 37°C temperature incubator for 24 h, the cells were inoculated on solid culture medium and cultured for 16–24 h.

#### 2.5.4 Colony forming units (CFUs)

Urushiol was dissolved in concentrations of 0.1, 0.5, and 1 wt%. The urushiol solutions were added in equal proportion to the bacterial suspension, and the culture was shaken for 24 h. Then, 100 μl of the 10-fold diluted solutions with sterile phosphate buffer (PBS, pH 7.2) was cultivated on BHI-agar plates and cultured for 48 h. The number of colonies was calculated.

#### 2.5.5 Biofilm cultivation

Three kinds of bacteria were anaerobically cultivated at 37°C for 24 h in 96-, 24- and 6-well plates.

#### 2.5.6 Crystal violet assay

After the removal of supernatant from the 24-well plate, 0.1% (w/v) Crystal Violet solution was injected into each well at room temperature, which was removed after 5 min. The cells were washed with PBS and DMSO was added to each well to dissolve the dye. Afterwards, cells were cultivated in the dark at room temperature with shaking for 30 min. Finally, absorbance was read at 570 nm using a microplate reader.

#### 2.5.7 Field emission scanning electron microscopy (FESEM)

FESEM was adopted to survey biofilm formation and the effects of urushiol on morphology. First, glass slices were UV-irradiated on each side for 2 h. Then, the glasses were laid in six-well plates, and a mixture of 1 ml *S. mutans, S. sanguinis*, and *S. gordonii* cell suspensions (10^5^ CFU/ml) and 1 ml urushiol solutions with concentrations of 0.1, 0.5, and 1 wt% were added to each well to form biofilms. After cultivation at 37°C for 24 h, the samples were fixed with 2.5% glutaraldehyde overnight at room temperature, dehydrated with ethanol solutions (50%, 70%, 90%, and 100%) for 30 min at each concentration, and dried overnight in a desiccator. The samples were observed by SEM with gold spray treatment at three randomly chosen points.

#### 2.5.8 Bacterial surface hydrophobicity assay

In short, the optical density (OD) of bacteria that were dispensed by centrifugation with 1.5 ml BHI was adjusted to OD_600nm_ = 0.5. The control and urushiol groups were set as described above. After cultivation under anaerobic conditions for 0 or 30 min at 37°C, the microtubes were centrifuged at 4°C for 5 min at 5,000 rpm, rinsed twice with sterile PBS, and resuspended in PBS. Absorbance was estimated at 550 nm (recorded as OD_1_). Subsequently, the microtube was shaken vigorously after the addition of 20% (v/v) xylene. Then, the mixture was allowed to stand until the aqueous phase was tested at 550 nm (recorded as OD_2_). The degree of hydrophobicity was determined using the following formula:

$$H=\left({OD}_{1}-{OD}_{2}\right){OD}_{2}\times 100\%$$

#### 2.5.9 Bacterial aggregation assay

Biofilm aggregation was measured using the 96-well plate method. After cultivation, three kinds of bacteria were resuspended in BHIS, and the OD value was adjusted to OD_600nm_ = 0.5. Then, the suspensions were injected into 96-well plates and the initial OD_600n_ value of each well was measured. The plates were then cultivated for 2 h at 37°C under anaerobic conditions. Afterwards, the equivalent of the upper suspension of bacteria was transferred to a new plate, and the absorbance of each well was measured at 600 nm. The extent of aggregation was calculated using the following formula:

$$A=\frac{\left({OD}_{initial}-{OD}_{2h}\right)}{\left({OD}_{initial}-{OD}_{blank}\right)}\times 100\%$$

#### 2.5.10 CCK8 assays

The biofilm samples (n = 9) were transferred to a new 96-well plate and cultivated at 37°C after injecting 10 µl CCK8 solution dropwise to each well to measure biofilm metabolic activity. After culture for 4 h in the dark, the absorbances of plates were recorded at 450 nm using a microplate reader.

### 2.6 The effects of urushiol etchants

#### 2.6.1 Atomic force microscopy (AFM)

In general, 20 dentin samples were subjected to AFM (n = 5). AFM assays were measured in tapping mode, and micrographs were chosen from selected areas of each surface randomly. The scan area of 5 × 5 µm was used to obtain height images and determine the average roughness (Ra) of the sample. The cross-sectional images and average value of the roughness were analyzed using Bruker NanoScope Analysis software.

#### 2.6.2 Contact angle analysis

After polishing with 1000-grit SiC sandpaper, the dentin slabs were rinsed twice for 10 min to remove residues. The specimens were treated with different etchants for 15 s and washed with deionized water for 15 s. An aliquot of 6 μl of deionized water was dropped onto the dentin surface by a contact angle meter (Data physics OCA20).

#### 2.6.3 SEM observations

Sound human third molars were cut perpendicularly to the crown-apex axis to expose the flat dentin surface. After polishing with 600-grit SiC sandpaper, the dentin substrates were etched with the aforementioned different experimental etchants for 15 s. Then, specimens were washed with deionized water for 10 s. The samples were dehydrated in ascending ethanol solutions (50%–100%), and 2.5% glutaraldehyde solution was used as the final fixation agent. After dehydration, samples were spray-coated with gold, and then observed under an SEM at 10 kV. The etchant-demineralized region was also analyzed by energy dispersive X-ray spectroscopy (EDS) coupled to SEM.

### 2.7 Microtensile strength test

#### 2.7.1 The microtensile bonding strength of dentin

Twenty non-carious human third molars were obtained from anonymous individuals after they provided informed consent. Enamel was removed using a low-speed diamond saw under flowing water to expose deep coronal dentin. To obtain a standardized smear layer, the dentin surface was polished with 600-grit wet silicon carbide sandpaper under cool water. Dentin etching was performed with 37% H_3_PO_4_ gel or experimental urushiol 10% phosphoric acid etchant. After dentin etching, a universal adhesive was applied on dentin in etch-and-rinse mode. universal adhesive was cured for 20 s following the manufacturer’s instructions. Subsequently, the resin composite was built up on the interface every 2 mm twice and light-cured for 40 s. This resulted in the establishment of three experimental groups. The components of adhesives and etchants, including their steps for use, are shown in Table 1.

**TABLE 1:** The components of the commercial and experimental products.

Product	Components	pH	Application procedures
Universal Adhesive	HEMA, MDP, Vitrebond copolymer, dimethacrylate resins, water, ethanol, initiators, and silane	2.7	1. After etching, apply adhesive on the dentin and shake for at least 20 s
			2. Air-dry by oil- and moisture-free compressed air until a smooth immovable film is obtained
			3. Light-curing with a LED light-curing unit for 20 s
Experimental etchant	Urushiol, water, DMSO, and phosphoric acid	4.5	1. Apply the etchant on the surface of the tooth allowing the urushiol and acid to interact with the tooth for 15 s
			2. Thoroughly wash off the etchant from the surface with water spray and dry with compressed air
			3. Carry on with the bonding steps
Commercial etchant	Phosphoric acid (37 wt% in water), color pigments, and thickening agent	0.4	The same as above

The specimens were stored in deionized water at 37°C for 24 h and then cut parallel to the long axis into small rods using a diamond saw under cool water with a cross-sectional area of 1.0 mm^2^. In conclusion, the microtensile strength (MPa) was the ratio of the maximum load to the area of the specimen.

#### 2.7.2 MMP inhibition tests

Dentin slabs were obtained in the same manner as with the μTBS test. Each slab was divided into two pieces further so that the four experimental groups were tested with the same dentin matrix. The tooth slices were glued onto the cover glass slide and polished to a thickness of 50 µm by wet silicon carbide sandpaper. For *in situ* zymography, self-quenched fluorescein-conjugated gelatin was used as the MMP substrate. Then, slices were incubated in a humidified chamber overnight at 37°C. After incubation, slices were examined using a confocal laser scanning microscope.

#### 2.7.3 The microtensile bonding strength of enamel

The buccal and lingual surfaces of the enamel were prepared. For the specimens, enamel surfaces were cut by slow-speed cutting under cool water and rinsed with SiC papers. In the same way as dentin bonding, enamel was bonded to the resin composite and cut for the test.

#### 2.7.4 Fracture mode

After collecting specimens that were tested for microtensile strength and dried naturally, a stereomicroscope was applied to observe the microstructure of the fracture interface and record the fracture mode. In summary, fracture modes of specimens are usually divided into three types: interface failure, mixed failure, and cohesive failure.

#### 2.7.5 Aging

Half of the bonded samples, including dentin and enamel specimens, were chosen randomly for 5,000 aging cycles (5°C for 30 s and 55°C for 30 s). The μTBS test was conducted as described above.

#### 2.7.6 CLSM of the interface

Samples of the dentin bonding interface specimens were bonded with 0.1% rhodamine B-added universal adhesive. Tests were performed with three slabs per group that were prepared in the same way as for the microtensile bond strength test.

### 2.8 Statistical analysis

SPSS (IBM SPSS Statistics 20, Armonk, NY, United States) was used for analyses. Data obtain from the μTBS test were analyzed using two-way (variables: different urushiol concentrations and aging) analysis of variance (ANOVA) and Tukey’s test. One-way ANOVA and Tukey’s test were used to calculate the other test results. Statistical significance was set at 0.001, 0.01, and 0.05.

## 3 Results

### 3.1 Dentin biostability

#### 3.1.1 Molecular docking test

**FIGURE 2:**
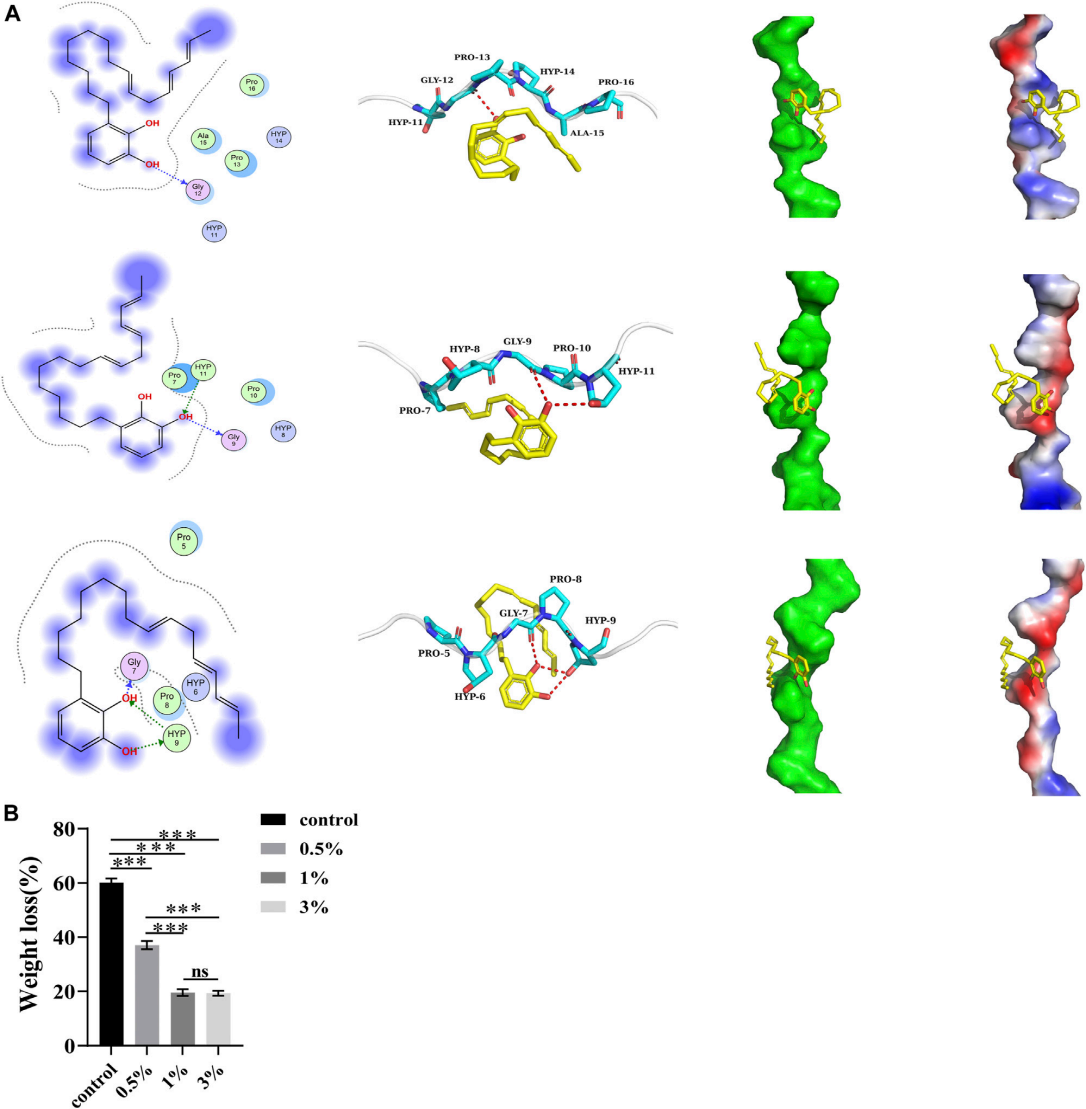
**(A)** Proteins binding to urushiol. Proteins are expressed in the form of blue ribbons and urushiol is expressed in the form of its chemical structure. **(B)** The weight loss of different groups. All statistical data are presented as mean ± standard deviation (SD) (n = 5; ***p < 0.001; ns, not significant).

Based on the binding modes of the target compound obtained by docking with 1cgd, 1qsu, and 4oy5 proteins, the left figure shows the two-dimensional interaction diagram (the dotted arrow in the figure indicates the hydrogen bond interaction), the middle figure shows the three-dimensional interaction diagram (the red dotted line in the figure indicates the hydrogen bond interaction), and the right figure shows the spatial complementarity diagram of small molecules and proteins (red in the figure indicates a negative charge, and blue indicates a positive charge) (Figure 2A).

Figure 2 shows the binding complexes and binding information of small molecule target compounds, with 1cgd, 1qsu, and 4oy5. The target compound binds to the helical surface of each protein. Two-dimensional and three dimensional interaction diagrams show that the target molecules and proteins mainly interact through hydrogen bonds. Among them, hydrogen bonds with the 4oy5 protein were more frequent, suggesting that the target molecule has the best binding with 4oy5. In addition, we noticed from the electrostatic surface diagram that the phenolic hydroxyl group of the molecule can always contact the charged surfaces of the three proteins, which should be conducive to the interaction between the target molecule and each protein.

A negative binding affinity indicates the possibility of binding. In this complex, the docking software generated binding affinity scores of −2.8 kcal/mol, −2.6 kcal/mol, and −2.2 kcal/mol for the target compound with 4oy5, 1cgd, and 1qsu proteins, respectively, indicating that the compound binds well with 4oy5, 1cgd, and 1qsu proteins.

#### 3.1.2 Collagenase biodegradation resistance

After etching, the endogenous collagenases are activated. As a result, biodegradation of dentine fibrils decomposes collagen molecules into small peptide fragments, which are lost. The ratio of mass loss of the control group was approximately 60.11%. This ratio was significantly higher than that of the urushiol groups (*p* < 0.05). In the urushiol groups, there was no significant difference (Figure 2B).

### 3.2 Antibacterial activity of urushiol

#### 3.2.1 Growth curves

**FIGURE 3:**
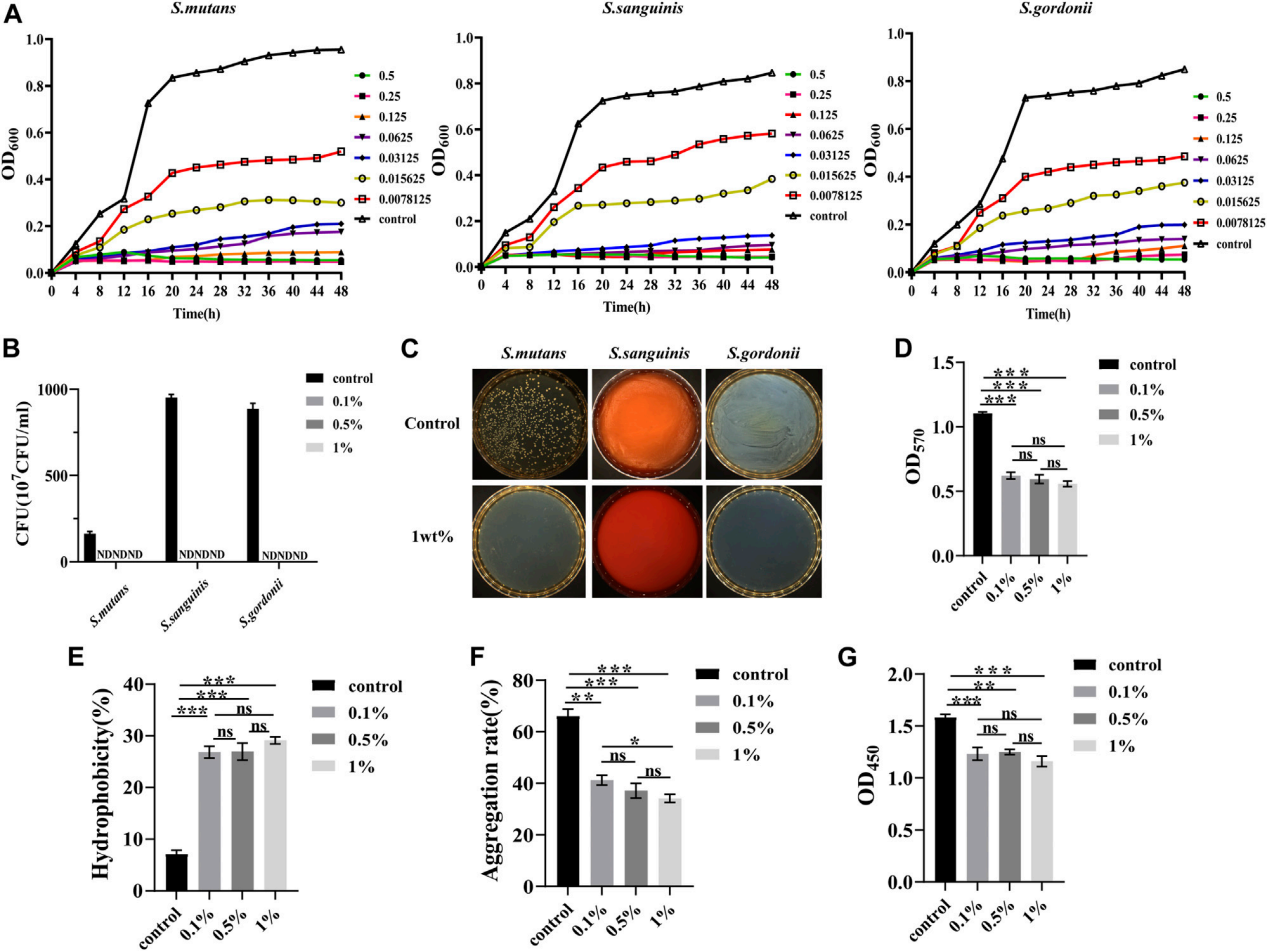
**(A)** Typical growth curves of the different concentrations of urushiol culture with *S.mutans*, *S.sanguinis*, and *S.gordonii*. **(B)** Colony forming units of the co-cultivation with urushiol and bacteria. (ND, not discovered). **(C)** Colony count units assays on solid culture medium. **(D)** The OD values at 570 nm of crystal violet assays. **(E)** The hydrophobicity percentage values of co-cultivation. **(F)** Aggregation rate percentages of co-cultivation. **(G)** The OD values at 450 nm in CCK8 assays. All statistical data are presented as mean ± standard deviation (SD) (n = 5; *p < 0.05; **p < 0.01; ***p < 0.001; ns, not significant).

In Figures 3B–D show that there were significant changes in the bacterial growth curves of the experimental groups, with an extended delay period. In the stable period, the antibacterial activity weakened with the extension of urushiol, indicating that increasing the concentration of urushiol higher than 31.25 μg/ml or 62.5 μg/ml did not significantly enhance the antibacterial effect. The levels of the three kinds of bacteria were significantly suppressed.

#### 3.2.2 MIC and MBC

The minimum inhibitory concentration (MIC) and minimum bactericidal concentration (MBC) of urushiol against *S. mutans, S. sanguinis*, and *S. gordonii* were measured by visual observation and plate coating (Table 2). After 48 h of anaerobic culturing at 37°C, the concentration without precipitation on the 96-well plate was the minimum inhibitory concentration as the urushiol concentration was 15.625 μg/ml, while no colonies grew on the solid plate with concentrations of 31.25 μg/ml in the *S. sanguinis* and *S. gordonii* groups and 62.5 μg/ml in *S. mutans* group.

**TABLE 2:** MICs and MBCs of urushiol against three kinds of bacteria.

Bacterial species	MIC (μg/ml)	MBC (μg/ml)
*S.mutans*	15.625	62.5
*S.gordonii*	15.625	31.25
*S.sanguinis*	15.625	31.25

MIC, minimal inhibitory concentration; MBC, minimum bactericidal concentration.

#### 3.2.3 Colony forming units

The calculation results of colony count and antibacterial rate are shown in Figures 3E, F. The antibacterial substance urushiol showed perfect antibacterial effects in the colony count. The colony count results demonstrated that differences among all groups were statistically significant (*p* < 0.05). When the concentration of urushiol was higher than 0.1 wt%, the antibacterial rate was mostly 100%.

#### 3.2.4 Crystal violet assay

Total biofilm activity and biofilm biomass were used to evaluate the effect of urushiol on fresh biofilm formation. As shown in Figure 3D, significant changes were observed between the control and urushiol groups (*p* < 0.05). Urushiol at 0.1wt% decreased the biomass significantly compared with that of the control group (*p* < 0.05). The results of this assay showed that there was an efficient effect of urushiol on biofilm formation.

#### 3.2.5 Bacterial surface hydrophobicity and aggregation assays

Significant changes were detected between the control and treatment groups (*p* < 0.05). Urushiol at the concentration of 0.5 wt% increased biofilm surface hydrophobicity (*p* < 0.05) (Figure 3E). Urushiol-treated groups showed effectively decreased bacterial aggregation (*p* < 0.05) (Figure 3F).

#### 3.2.6 CCK8

The results of the CCK8 test are shown in Figure 3G. The urushiol-treatment effectively decreased the metabolic activity of biofilms (*p* < 0.05).

#### 3.2.7 SEM

**FIGURE 4:**
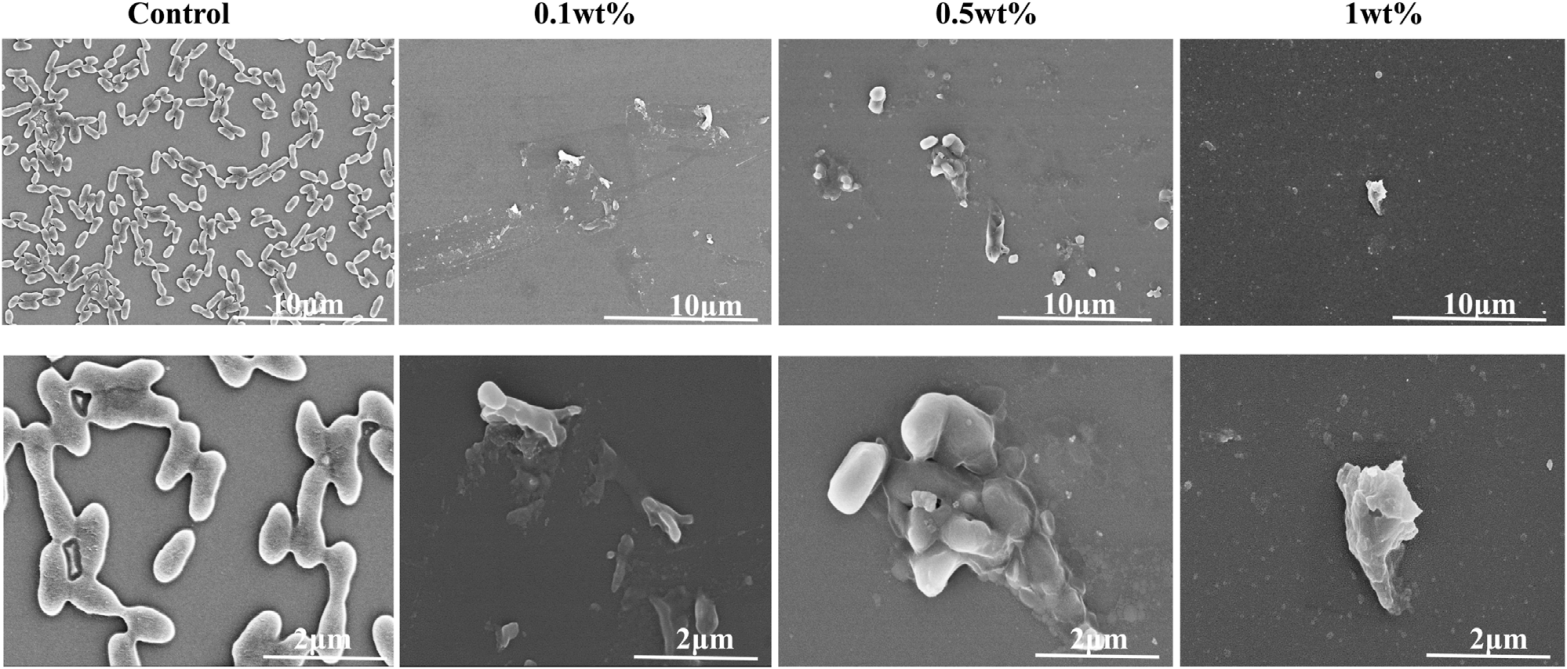
SEM examination of the co-cultivation of different concentrations of urushiol with biofilm.

Compared with the control group, FESEM micrographs (Figure 4) showed that urushiol treatment inhibited bacterial growth, although at low concentrations. As the concentration increased, the biofilm became thinner and the morphology of the bacterial cell was disrupted with the damaged cell membrane.

### 3.3 AFM

The three-dimensional images of atomic force microscopy showed that the dentin surface in the control group was rougher than that in the urushiol treatment groups. However, the roughness of the dentin after etching increased with increasing concentrations of urushiol (*p* < 0.05). Consistent with the morphology results, the Ra values were also highest in the treated group (*p* < 0.05).

### 3.4 SEM of the dentin after etching

**FIGURE 5:**
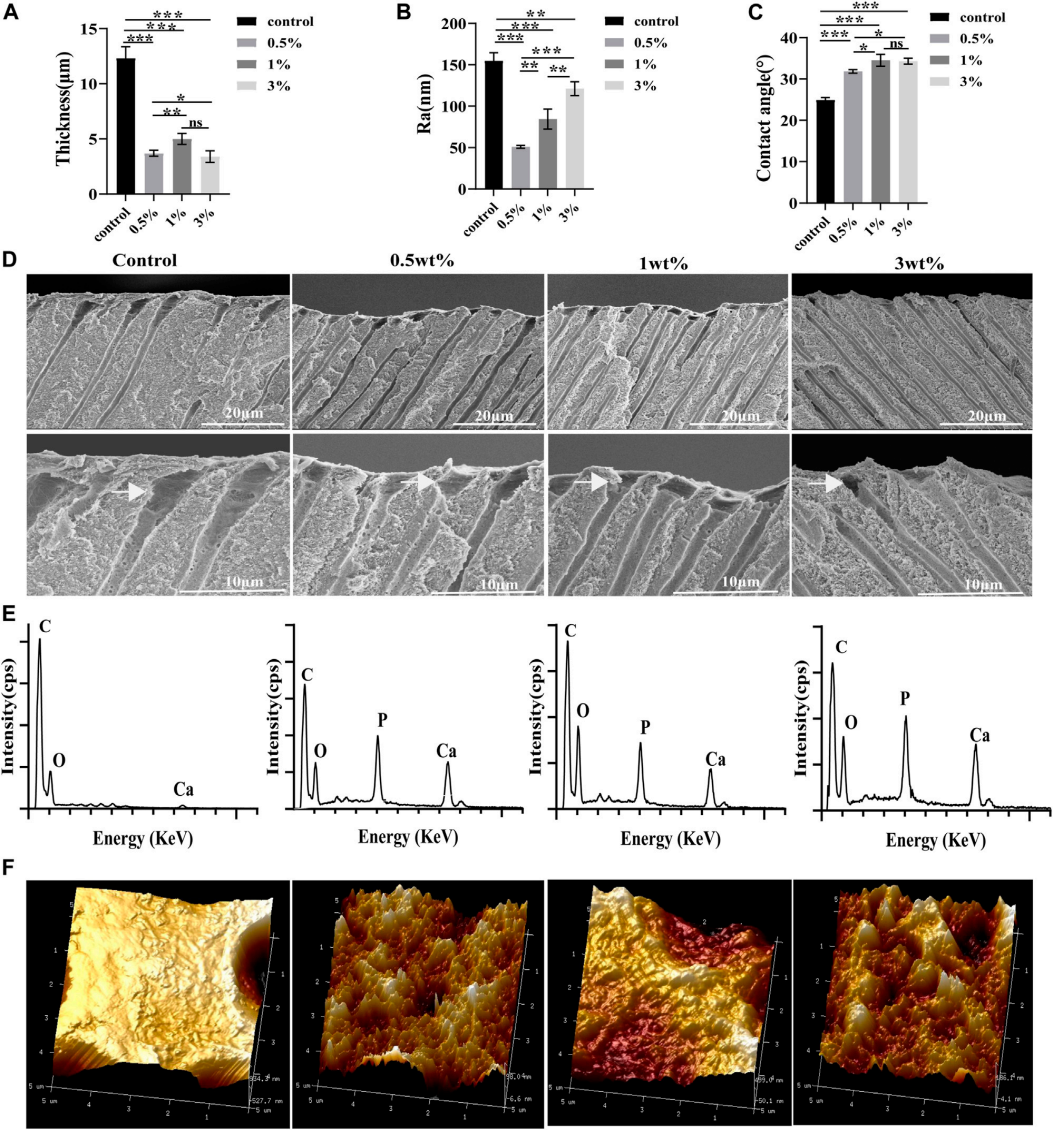
**(A)** The etching values of different etchants. **(B)** The Ra values of the dentin surface after etching. **(C)** Contact angle values of the dentin surface after etching. **(D)** SEM images of the dentin parallel to the tooth long axis after etching. **(E)** EDS point analysis for the elements of the etching surface. **(F)** Atomic force microscope analysis of the roughness of dentin after etching. All statistical data are presented as mean ± standard deviation (SD) (n = 5; *p < 0.05; **p < 0.01; ***p < 0.001; ns, not significant).

The etching depth in the control group was approximately 12.33 µm in Figure 5, while the 1% urushiol-modified etchant had a depth of approximately 2–4 μm, which was similar to the etching effect of mild-acidic self-etching adhesives to some extent. The collagen fiber on the surface collapsed after acid etching and the elements were measured by the EDS spectra. It was demonstrated that the control group removed both phosphate and calcium elements from dentin, while dentin etched by biomodified etchant displayed the presence of remnant calcium.

### 3.5 Contact angle analysis

Compared with those of the control group, contact angles of the dentine surfaces after acid etching by the urushiol-modified etchants were significantly higher (*p* < 0.05). Urushiol etchant (1 wt%) effectively improved the hydrophobicity of the dentine surface (*p* < 0.05).

### 3.6 μTBS test

Bond strength results obtained instantly and over time are listed in Table 3. In the bonding experiment, the bonding strengths of dentin and enamel increased with increasing urushiol concentrations, but there was no significant difference between the 1 wt% and 3 wt% urushiol groups. Compared with those of the control group, the bonding strengths of the experimental groups significantly increased, and there were smaller reductions in bonding strengths after aging. After aging thermocycles, the bond strength significantly declined in all groups (*p* < 0.05) except for the 1 and 3 wt% urushiol etchant groups. The bonding strength of enamel was higher than that of dentin in the control group; however, the μTBS of urushiol-treated groups was lower than that of the control group in enamel bonding. The aging process had less of an effect on enamel (*p* < 0.05).

**TABLE 3:** μTBS of the urushiol and control groups obtained instantly (immediate) and over time (aging) (MPa, n = 9, mean ± SD).

Group	Dentin		Enamel	
	Immediate	Aging	Immediate	Aging
Urushiol-0.5%	36.23 ± 0.33^Ab^	33.93 ± 0.62^Bb^	35.25 ± 0.63^Ca^	30.22 ± 1.75^Da^
Urushiol-1%	41.31 ± 1.24^Ac^	39.34 ± 0.57^Bc^	34.22 ± 1.86^Ca^	30.63 ± 2.69^Da^
Urushiol-3%	41.49 ± 1.97^Ac^	40.84 ± 2.78^Ac^	33.92 ± 2.65^Ca^	31.53 ± 1.79^Da^
Control	29.06 ± 1.33^Aa^	24.19 ± 0.71^Aa^	33.92 ± 2.65^Ca^	30.89 ± 1.76^Ca^

Different lowercase letters in the same column imply statistically significant differences (*p* < 0.05; vertical comparisons). Different capital letters in the same line imply statistically significant differences (*p* < 0.05; horizontal comparisons).

### 3.7 Fracture mode

The control group was mainly characterized by interfacial fracture and the experimental groups mainly exhibited mixed failure. After aging, there was no significant change in the main fracture mode of each group of specimens, but the proportion of interfacial fracture increased.

### 3.8 Gelatin zymography

**FIGURE 6:**
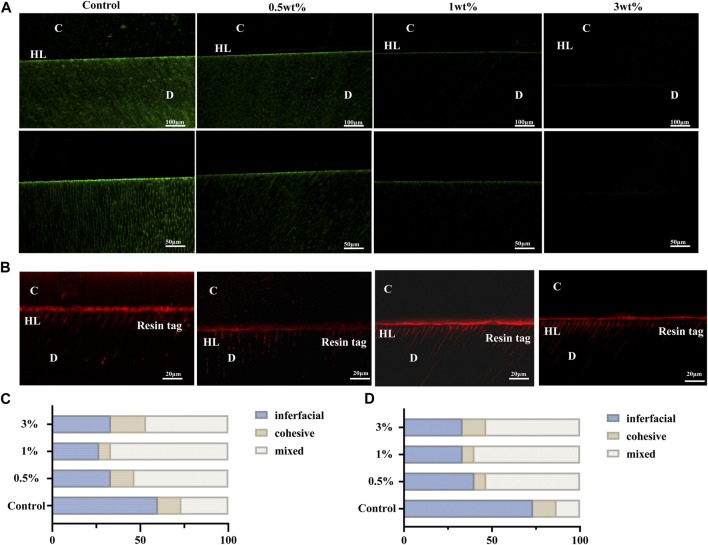
**(A)** Typical images of the gelatin morphology of each group. **(B)** Confocal laser scanning microscope images of the bonding interface of dentin.**(C)**Fracture mode in the immediate bonding test. **(D)** Fracture mode in the aging bonding test. C, composite resin; D, dentin; HL, hybrid layer.

The etching process will activate the MMPs in dentin. As shown in Figure 6A, the green fluorescence part was the activated MMPs in dentin. In the control group, the etching process could obviously activate the MMPs in the hybrid layer and dentinal tubules as the areas of green fluorescence. However, in the experimental groups, the green part was mainly concentrated in the hybrid layer, with a relative shallow depth and weaker light intensity, indicating that urushiol could effectively inhibit the activities of MMPs.

### 3.9 Morphology of resin-dentin interfaces

As shown in Figure 6B, the red fluorescence was Rhodamine B added in the adhesives. Rhodamine B could penetrate the hybrid layer and the dentinal tubules with the adhesives, so the part that emitted red fluorescence could show the depth of the penetration of the adhesives. The hybrid layer and resin tags are the main influencing factors of the bonding strength. Compared with the control group, after treatments with urushiol etchants, the hybrid layer and resin tags displayed denser and thicker images. This was consistent with the experimental results of bonding strengths.

## 4 Discussion

Degradation of the hybrid layer is mainly caused by intrinsic enzymes and bacteria (Pashley et al., 2004; Frassetto et al., 2016). Many studies have added bioactive components, such as collagen crosslinkers or MMP inhibitors into primers, but this process requires extra clinical steps (Maravic et al., 2018; Beck and Ilie, 2022). However, in this study, we applied the natural bioactive urushiol obtained from lacquer sap into phosphoric acid as a novel etchant. The results indicated that the novel etchant could improve the biostability of dentin and kill bacteria at low concentrations. As a result, the dentin bonding strength increased without additional steps.

The biomodified etchant with urushiol applied in this study may be defined as an intermediary-strong etching strategy. Although adhesives have been developed for many generations, the etch-and-rinse mode is the gold standard (De Munck et al., 2011). The hybrid layer of the E&R mode is thicker than that of the mode without etching. Moreover, with the universal adhesive applied in other experiments, the etch-and-rinse mode exhibited the highest bonding strength (Takamizawa et al., 2019). Therefore, the etchant was essential to dentin and enamel bonding. In this study, urushiol is a natural monomer addition, which has been proven to effectively improve collagen crosslinking and exhibit antibacterial properties. As an effective bioactive component in the etchant, its performance was tested in this study.

The etching process can lead to the exposure of dentin collagen in the hybrid layer and activate endogenous enzymes. The enhancement of collagen biostability is beneficial for promoting dentin bonding performance (Bedran-Russo et al., 2008). The molecular docking results indicate the existence of hydrogen bonds when urushiol approaches the collagen fibril. And in the collagenase test, weight loss caused by endogenous enzymes decreased with urushiol treatment. The dentin biostability results above suggested that urushiol can enhance collagen biostability at low concentrations. Such a phenomenon may be regarded as adjunctive evidence for hydrogen bonds. Moreover, the gelatinolytic activity of the MMPs within the hybrid layers etched with urushiol etchants were inhibited to varying degrees, which may be attributed to the coupling effect between catechol and the Ca^2+^ site of the protease (Visse and Nagase, 2003).

Dental caries has been studied generally for hundreds of years. Among cariogenic factors, *S. mutans* and its biofilm formed on tooth surfaces are the major factors (Zhang et al., 2022b). In recent years, the antimicrobial properties of plant extracts have attracted widespread attention. *S.gordonii* and *S.sanguinis* can inhibit *S.mutans* by H_2_O_2_ and are considered anti-caries probiotic bacteria (Kuramitsu and Wang, 2006). Therefore, the multispecies biofilm is more conformed to the local microecology of the oral cavity. The MIC and MBC tests showed that 15.125 μg/ml of urushiol could effectively inhibit the three kinds of bacteria. When the concentration of urushiol was 0.1 wt%, almost no colonies could be seen in colony forming unit tests. Adhesion and aggregation are prerequisites for biofilm formation. Interestingly, treatment with 0.5 wt% urushiol improved the hydrophobicity of the biofilm, and the adhesion of bacteria decreased accordingly, which was beneficial for inhibiting the formation and maturation of carious biofilms. Scanning electron microscopy experiments showed that the bacterial biofilm on glass sheets was thicker in the control group, proving the antibacterial activity. Moreover, the metabolisms of biofilms in CCK8 assays significantly decreased after treatment with urushiol.

The μTBS tests showed that the urushiol etchants can effectively improve dentin bonding, which cause by the dentin biostability with instinctive antibacterial property. In orthodontics, the quantity and quality of etched patterns produced by acids play an important role in the wettability and contact angle between the adhesive and enamel surface. The better the etching pattern is, the more surface energy the enamel contains and the better the permeability of the adhesive, which ultimately leads to stronger bonding (Gardner and Hobson, 2001). Laser confocal observations found that the interfaces of urushiol groups had perfect hybrid layers and resin tags. The addition of a bioactive natural monomer to the etchant was evaluated to improve the durability of dentin bonding with the selected universal adhesive. However, all groups showed decreases in bond strength (Table 2). The results demonstrated that urushiol can effectively promote the biological activity of acid etchant and promote dentin bonding. However, there was no significant effect on the bonding strength of enamel with urushiol etchant treatment.

To combat infections, two effective strategies have been investigated. One is to deactivate bacteria by adding various antimicrobial materials (Zhang et al., 2022a). The other is to regulate the microbial balance without antibacterial substances (Harimoto et al., 2022; Lu et al., 2022). As an environment-friendly and renewable natural material with good antimicrobial effects, urushiol, mostly extracted from lacquer sap, exhibits a range of biological activities, such as antibacterial, antioxidant, antiviral, and immunological properties (Kim et al., 2014). Urushiol exerts an extensive antibacterial effect on Gram-positive and Gram-negative bacteria (Jie et al., 2023). It has been shown that urushiol additionally provokes cell membrane damage of bacteria and deactivates enzymes involved in fatty acid biosynthesis, limiting the production of toxic bacterial metabolites with its phenolic hydroxyl radicals and unsaturated alkyl chains (Suk et al., 2010). Therefore, our study incorporated 0.5–3 wt% urushiol into etchants and found that it not only improved the biostability of dentin but also killed bacteria effectively. Accordingly, the urushiol groups could significantly increase bond strengths initially and over time. Urushiol may be an attractive candidate for use in future medical applications.

## 5 Conclusion

Urushiol not only improves dentin biostability but effectively reforms the oral microecology. As a result, urushiol etchant can enhance the interface of bonding to obtain a superior bonding strength with enamel and dentin. Therefore, urushiol may have promising applications in dentin adhesion.

**SCHEME 1:**
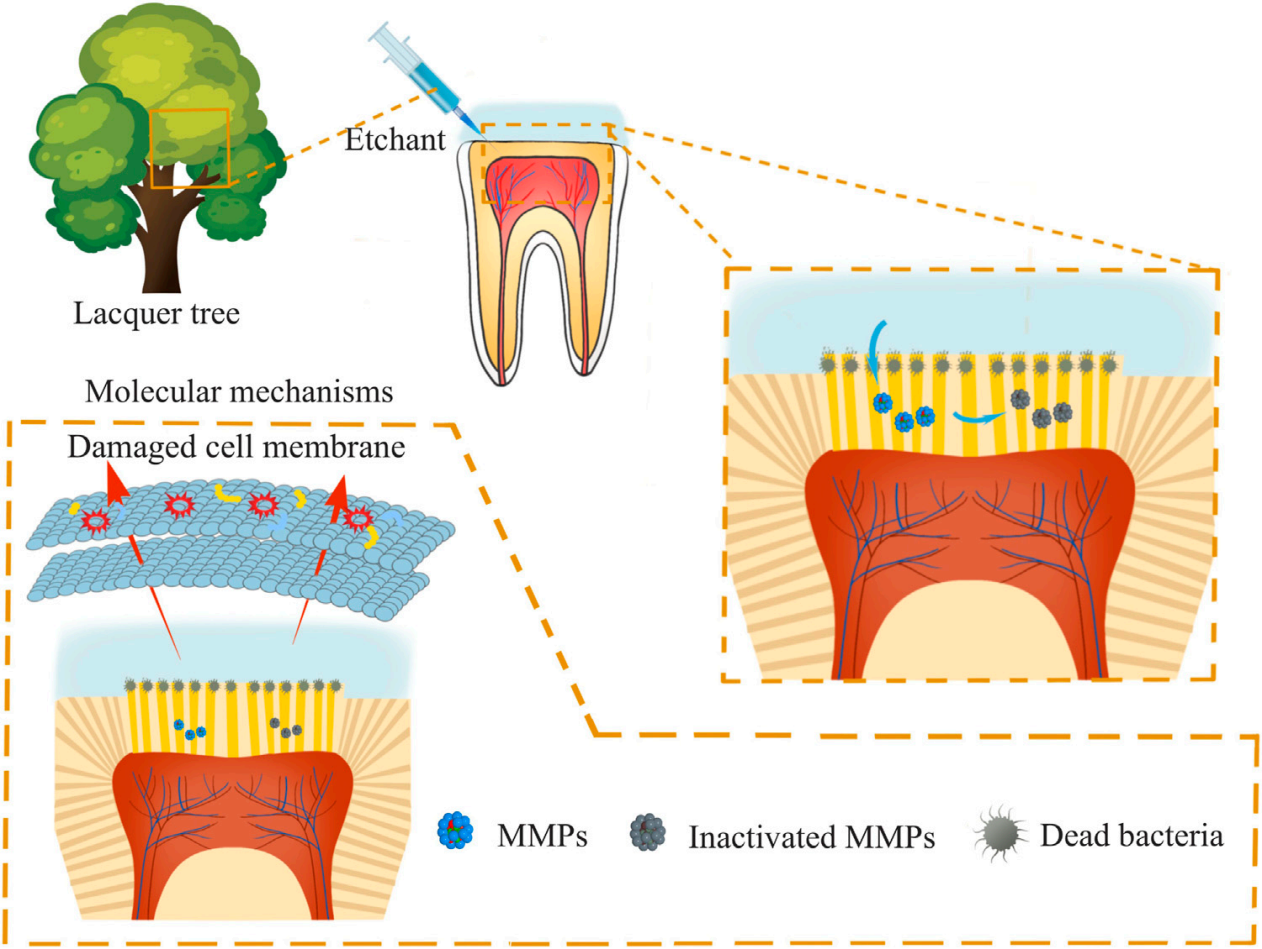
Schematic diagram of dentin surface applied with urushiol etching.

## Data Availability

The original contributions presented in the study are included in the article/Supplementary material, further inquiries can be directed to the corresponding author.
